# An open-label phase I dose-finding study of APR-246 in hematological malignancies

**DOI:** 10.1038/bcj.2016.60

**Published:** 2016-07-15

**Authors:** S Deneberg, H Cherif, V Lazarevic, P-O Andersson, M von Euler, G Juliusson, S Lehmann

**Affiliations:** 1 Karolinska University Hospital, Center of Hematology and Karolinska Institutet, Institution of Medicine, Huddinge, Sweden; 2 Department of Medical Sciences, Uppsala University and Uppsala Academic Hospital, Uppsala, Sweden; 3 Department of Hematology, Skane University Hospital, Lund, Sweden; 4 Section of Hematology, South Älvsborg Hospital, Borås and Sahlgrenska Academy, Gothenburg, Sweden; 5 Aprea AB, Solna, Sweden

Mutations in the *TP53* tumor suppressor gene occur at different frequencies in a variety of human malignancies.^1^ *TP53* encodes a DNA-binding transcription factor that induces cell growth arrest, senescence and cell death by apoptosis upon cellular stress, including oncogenic stress and DNA damage. In hematological malignancies *TP53* mutations are common in relapsed/refractory disease and confers a dismal prognosis.^2^ PRIMA-1 and the analog APR-246 (PRIMA-1^MET^) can restore wild-type conformation of mutant p53 and induce apoptosis in tumor cells of various origin.^3, 4, 5^ APR-246 is the first compound targeting mutant p53 to enter into a clinical trial. In the first in-man study APR-246 was given daily as a 2 h intravenous infusion for 4 consecutive days. The maximum tolerable dose using this schedule was defined as 60 mg/kg daily and the most common adverse effects were neurological.^3^ This is an extension of the first in-man study aiming at optimizing the dose regimen to obtain better antitumor response with less toxicity.

The primary objective was to evaluate safety and tolerability of APR-246 with the new dosing schedule. Secondary objectives were to determine the pharmacokinetic (PK) profile and assess antitumor effects of APR-246.

Main inclusion criteria were: relapsed/refractory non-M3 acute myeloid leukemia (AML) or chronic lymphatic leukemia (CLL) with deletion of 17p not eligible for other therapies, age ⩾18 years, ECOG performance status 0–2 and life expectancy of at least 2 months. Main exclusion criteria were hepatic transferase ⩾5 upper limit of normal (ULN), creatinine ⩾1.5 ULN, severe cardiac or respiratory disease. The trial was conducted in accordance to the Declaration of Helsinki and ICH-GCP guidelines and was registered at ClinicalTrials.gov, identifier NCT00900614.

The dosing schedule was designed to maximize drug exposure without increasing peak concentrations. Four daily infusions were given using a boosting infusion of 50 mg/kg during 45 min followed by a 85 mg/kg as a 5 h and 15 min infusion, in total 135 mg/kg, with an option to repeat the treatment in 21 day cycles. Interim analyses were scheduled at completion of every three patients or whenever necessary at any occasion. Dose limiting toxicity (DLT) was defined as study drug–related Common Terminology Criteria for Adverse Events (CTCAE) grade 1 for ataxia/incoordination, tremor and confusion; CTCAE grade 2 for somnolence, depressed level of consciousness and seizures; and other CTCAE grade 2, 3 or 4. Dose de-escalation was made twice by decision from the study board due to toxicity. After treating three patients on the starting dose of 135 mg/kg, there was a dose reduction to 105 mg/kg (45 mg/kg bolus+60 mg/kg infusion). This schedule was given to five patients when a second de-escalation was made to 67.5 mg/kg (25 mg/kg+42.5 mg/kg). Efficacy was assessed by blood and bone marrow sampling at baseline, on day 4 and on day 21 for AML, and by lymph node evaluation manually; and with computed tomography (CT) scans at baseline and on day 21 for CLL. Predefined response criteria for AML was a reduction of blast cell count of ⩾25% in peripheral blood and/or bone marrow, and for CLL a reduction of lymphocytes in peripheral blood of ⩾25% and/or a ⩾25% reduction of tumor size assessed by CT scan or palpation of peripheral lymph nodules and/or disappearance of B symptoms.

A Mini Mental Test was performed before and after infusion at day 1 and day 4. Safety visits twice weekly were made until day 21. PK samples were collected at baseline, 45 and 90 min after start and 2 h after completed infusion. PK calculations were performed by non-compartmental analysis. Plasma concentrations of APR-246 were determined using a validated LC-MS/MS method.^3^

Eleven patients were screened and ten were included at four centers, eight with AML and two with CLL. One patient failed screening due to an ongoing systemic infection and was not included. All patients had relapsed or refractory disease with a median of two previous lines of therapy (range 1–3). Three out of eight patients analyzed (exon 2–11) had *TP53* mutations. Patient demographics are summarized in Table 1.

Using the 6 h infusion administration, the PK of APR-246 was characterized by a low clearance (151±35 ml/h per kg) and a large distribution volume (824±219 ml/kg; mean±s.d., *n*=10) indicating a distribution of APR-246 throughout the whole body. APR-246 concentrations declined in plasma with a *t*_1/2_ of about 3.8±0.8 h. At steady state (day 4), no accumulation of APR-246 was observed with the adopted 6 h infusion schedule or after repeated cycles. Plasma clearance and distribution volume were similar at day 1 and day 4, suggesting time-independent kinetics, and in line with the PK parameters previously obtained in the range of doses 2–90 mg/kg after a 2 h infusion. This suggests dose-independent kinetics also up to a daily dose of 135 mg/kg per day given as a 6 h infusion.

In total, 41 adverse events (AE) were considered to have probable or possible relationship to study treatment. There was a relation between the dose and the number of AE:s probably associated with study medication: 16, 11 and 1 AE:s were reported at dose levels 135, 105 and 67.5 mg/kg, respectively (Table 2). The most commonly reported AE were vomiting, constipation and dizziness, followed by nausea, electrocardiogram QT-prolongation, cough and fatigue.

Two AE:s were classified as dose limiting at 135 mg/kg; dizziness, reported for the second included patient, and tremor, reported for the third included patient. Based on this, the dose for the following patients was decreased to 105 mg/kg.

The only two severe adverse events (SAE) judged as probably related to the study medication occurred at this dose level, both in the same patient (03-102) and on the same day; electrocardiogram QT-prolongation and dyskinesia. Patient 03-102 was discontinued from the study due to the events and a second dose reduction to 67, 5 mg/kg was made in accordance to the pre-specified DLT criteria. No DLT or SAE:s were reported at that dose level.

Since QT-prolongation was previously unreported for APR-246 and due to its potential significance, a retrospective independent expert review of all electrocardiogram (ECG:s) was undertaken. Of note, the review showed no evidence of consistent, systematic, clinically significant ECG changes after exposure to APR-246, including for patient 03-102, partly since some patients had prior cardiac morbidity that is not amenable to QTc evaluation, for example, atrial fibrillation. However, a minor QT-prolongation could not be ruled out and ECG:s in triplicate and exclusion of patients with grade 1 QTc-prolongation is advised in future studies.

Two patients fulfilled the predefined response criteria at day 21. One CLL patient (01-101) receiving treatment at 135 mg/kg showed a reduction of the lymphocyte count >25% and a >25% reduction in lymph node size between day 1 and day 4. This patient received two additional treatment cycles at the 105 mg/kg dose, but no further responses were seen and the patient progressed during cycle 2. One AML patient (07-103, 67.5 mg/kg dose) showed a reduction of blast cell count in peripheral blood with >25%, however, the patient was not given any additional treatment due to experiencing SAEs (judged as unrelated to the study treatment) at the end of the follow-up period. The patient progressed and died 29 days after start of treatment.

A third patient (03-101), a previously stem cell-transplanted AML patient in third relapse, was treated with 135 mg/kg APR-246 and went into complete remission. A month prior to inclusion the patient had 83% blasts in peripheral blood (judged as overt relapse and no bone marrow was taken) compared with 4% after 4 days of APR-246 treatment and 0% at the day 21 bone marrow examination. However, the role of APR-246 for this remarkable response was not possible to interpret due to concurrent treatment with hydroxyurea (500–1000 mg q.d.) and sorafenib (200 mg b.i.d) initiated 4 and 3 weeks preceding study medication, respectively. This paired with a missing baseline evaluation due to dry tap and severe pancytopenia precluded proper evaluation. The patient remained in complete remission with incomplete platelet recovery (CRi) for 3 months maintaining sorafenib and hydroxyurea until progression and the patient passed away during a second stem cell transplantation. No further treatment with APR-246 was given.

Noticeable, both patients who fulfilled the response criteria were *TP53* mutated. Taken together with the clinical efficacy data from the first in-human study, 5/6 patients with confirmed *TP53* mutations showed some sign of clinical activity.

In conclusion, the results of the extended study of APR-246 in patients with AML and CLL suggest that a dose regimen of 67.5 mg/kg, given as a 6 h infusion on 4 consecutive days is safe and well tolerated and that APR-246 should be further explored for hematological indications, preferentially in combination with standard chemotherapy.

## Figures and Tables

**Table 1:** Patient characteristics

*All patients*	n	10
Age	Median (range)	63.6 (32–78)
Gender	Male/female	6/4
		
*AML patients*	n	8
Prior therapies		
1/2/3 lines	*n*	3/2/3
*De novo*/secondary AML	*n*	6/2
* *Cytogenetics		
Low/intermediate/high risk	*n*	1/4/3
TP53 mutations	*n*(*n* analysed)	2(6)
		
*CLL patients*	n	2
* Prior therapies*		
2/3 lines	*n*	1/1
* FISH*		
del 17p	*n*	2
del 11q	*n*	1
TP53 mutations	*n*(*n* analysed)	1(2)

Abbreviations: AML, acute myeloid leukemia; CLL, chronic lymphatic leukemia; FISH, fluorescence *in situ* hybridization.

**Table 2:** Adverse events (AE:s) judged by investigators to be possibly or probably related to APR-246-administration

*Dose level*	*135 mg/kg 3 courses*		*105 mg/kg 7 courses*		*67.5 mg/kg 2 courses*	
	*Severe (CTCAE 3-4)*	*Moderate, mild (CTCAE 1-2)*	*Severe (CTCAE 3-4)*	*Moderate, mild (CTCAE 1-2)*	*Severe (CTCAE 3-4)*	*Moderate, mild (CTCAE 1-2)*
*Gastrointestinal*						
Vomiting		2 (67)		2 (29)		
Constipation				1 (14)		1 (50)
Nausea		2 (67)		2 (29)		
*Nervous system*						
Dizziness	1 (33)	1 (33)		2 (29)		1 (50)
Tremor		2 (67)		1 (14)		
Somnolence		1 (33)				1 (50)
Headache		1 (33)				
Lethargy		1 (33)				
Ataxia				1 (14)		
Dyskinesia			1 (14)			
*Investigations*						
Electrocardiogram QT prolonged		1 (33)	1 (14)	3 (43)		
Hemoglobin decreased				1 (14)		
*General*						
Fatigue		1 (33)	1 (14)	1 (14)		
*Thoracic*						
Cough		1 (33)				
*Musculoskeletal*						
Osteonecrosis	1 (33)					
*Ear and labyrinth*						
Vertigo		1 (33)		1 (14)		
*Metabolism*						
Hypokalemia		1 (33)				
*Psychiatric*						
Confusional state				1 (14)		
Disorientation			1 (14)			
*Vascular*						
Thrombophlebitis				1 (14)		

Number of AE:s are indicated with percentages in parenthesis.
